# Cell-type specific differences in promoter activity of the ALS-linked ***C9orf72*** mouse ortholog

**DOI:** 10.1038/s41598-017-05864-2

**Published:** 2017-07-18

**Authors:** Abraham J. Langseth, Juhyun Kim, Janet E. Ugolino, Yajas Shah, Ho-Yon Hwang, Jiou Wang, Dwight E. Bergles, Solange P. Brown

**Affiliations:** 1 Solomon H. Snyder Department of Neuroscience, Johns Hopkins University School of Medicine, Baltimore, Maryland 21205 USA; 2 Department of Biochemistry and Molecular Biology, Bloomberg School of Public Health, Johns Hopkins University, Baltimore, Maryland 21205 USA

## Abstract

A hexanucleotide repeat expansion in the *C9orf72* gene is the most common cause of inherited forms of the neurodegenerative disease amyotrophic lateral sclerosis (ALS). Both loss-of-function and gain-of-function mechanisms have been proposed to underlie this disease, but the pathogenic pathways are not fully understood. To better understand the involvement of different cell types in the pathogenesis of ALS, we systematically analyzed the distribution of promoter activity of the mouse ortholog of *C9orf72* in the central nervous system. We demonstrate that *C9orf72* promoter activity is widespread in both excitatory and inhibitory neurons as well as in oligodendrocytes and oligodendrocyte precursor cells. In contrast, few microglia and astrocytes exhibit detectable *C9orf72* promoter activity. Although at a gross level, the distribution of *C9orf72* promoter activity largely follows overall cellular density, we found that it is selectively enriched in subsets of neurons and glial cells that degenerate in ALS. Specifically, we show that *C9orf72* promoter activity is enriched in corticospinal and spinal motor neurons as well as in oligodendrocytes in brain regions that are affected in ALS. These results suggest that cell autonomous changes in both neurons and glia may contribute to C9orf72-mediated disease, as has been shown for mutations in superoxide dismutase-1 (SOD1).

## Introduction

A hexanucleotide repeat expansion mutation in *chromosome 9 open reading frame 72* (*C9orf72*) is the most common known genetic cause of amyotrophic lateral sclerosis (ALS), a fatal neurodegenerative disease characterized by the progressive loss of corticospinal, brainstem and spinal motor neurons. A *C9orf72* mutation underlies approximately 40% of familial and 5% of sporadic ALS cases^1–3^. This hexanucleotide expansion is also found in approximately 10% of cases of a second neurodegenerative disease, frontotemporal dementia (FTD), a common cause of dementia in middle-aged patients^3–5^. Mutations in *C9orf72* are a rare risk factor for several additional neurologic and psychiatric disorders including Alzheimer’s disease, Parkinson’s disease, Huntington’s disease phenocopy patients, multiple system atrophy, depressive pseudodementia, bipolar disorder, and schizophrenia^6–16^. However, it is not known how this genetic mutation leads to these cell type-specific neurodegenerative disorders.

Both loss-of-function and gain-of-function mechanisms have been proposed to mediate *C9orf72*-linked ALS^17, 18^. Several studies have demonstrated decreased *C9orf72* transcript and protein levels in patients with ALS and FTD^19–23^. Deletion of the mouse ortholog of the *C9orf72* gene (3110043O21Rik, referred to here as *C9orf72*) has been reported to shorten lifespan and induce modest motor deficits in some, but not all mouse models, and cause profound dysregulation of the immune system^24–29^. Gain-of-function toxicity of the repeat expansion induced by sense and anti-sense RNA transcripts, as well as dipeptide proteins generated through repeat-associated non-ATG (RAN) translation, are also thought to contribute to neurodegeneration^17, 18^. Several transgenic mouse lines recently developed using patient-derived gene constructs demonstrate that *C9orf72* repeat expansions induce age-dependent accumulation of RNA foci and dipeptide repeat proteins, along with neurodegeneration and behavioral abnormalities that at least partially recapitulate human disease^26, 28, 30, 31^. Although the complex mechanisms underlying *C9orf72*-related disease have not been resolved, understanding the expression pattern of *C9orf72* in the central nervous system (CNS) is not only important for understanding the pathogenesis of ALS, but is also relevant to the wide spectrum of *C9orf72*-associated diseases.

Although the signature of ALS is the loss of corticospinal, brainstem, and spinal motor neurons, multiple cell types have been shown to contribute to the pathogenesis of the disease. Non-neuronal cells including oligodendrocytes, astrocytes, and microglia are also critical players in the pathogenesis of ALS^17^. Although some progress has been made in understanding the cell-type specific expression of *C9orf72* ^26, 28, 32^, a comparison of the distribution of *C9orf72* expression across different neuronal and glial cell types in relevant regions of the brain and spinal cord is still lacking. Whether *C9orf72* promoter activity is specifically enriched in affected corticospinal neurons, spinal motor neurons, or oligodendrocytes in regions implicated in ALS is not yet fully understood.

Here, we systematically mapped the promoter activity of the mouse ortholog of *C9orf72* in a genetically engineered strain of mice containing a targeted *LacZ* insertion under the control of the *C9orf72* native promoter. Through quantitative comparisons among different types of neurons and glial cells labelled with retrograde neuronal tracers and cell type-specific markers, we demonstrate that mouse *C9orf72* promoter activity, although widespread throughout the brain and spinal cord, is specifically enriched in corticospinal and spinal motor neurons and in oligodendrocytes, subsets of cells known to undergo degeneration in ALS, in regions affected by ALS. In contrast, *C9orf72* promoter activity was detected in only a small percentage of microglial cells and even fewer astrocytes. Thus, these data suggest that, despite widespread expression, *C9orf72* promoter activity reflects the patterns of degeneration typically seen in this disease, consistent with direct cell autonomous toxicity.

## Results

### The distribution of *C9orf72* promoter activity and cellular density are highly correlated in the CNS

Mice have a single gene, 3110043O21Rik (here referred to as *C9orf72*), which is orthologous to human *C9orf72* ^32^. To determine the distribution of *C9orf72* promoter activity in the central nervous system, we generated chimeric mice using several mouse embryonic stem cell lines heterozygous for an allele with a *LacZ* insertion in the *C9orf72* locus generated by the Knock Out Mouse Project (KOMP)^29, 32–35^. The *LacZ* insertion results in deletion of exons 2–6 of the mouse *C9orf72* gene, producing a knockout allele (Supplementary Fig. 1a). Analysis of RNA-sequencing (RNA-seq) data from mice generated using the same targeting cassette and embryonic stem (ES) cell background indicates that the transcript structure is largely maintained between wild type (WT) and *C9orf72* ^*LacZ*/+^ mice^28^ (Supplementary Fig. 1b). Although WT transcripts have two alternative starts, exon 1a or 1b, while the *LacZ* allele appears to use only exon 1a, there is no reported evidence for differential usage of exon 1a and 1b among different cell types indicating that the *LacZ* reporter likely reflects the pattern of the wild type gene expression. The heterozygous mice have a normal phenotype until six months of age, after which a fraction of *C9orf72* ^*LacZ*/+^ mice exhibit an age-dependent decrease in survival, with approximately 20% of the heterozygotes dead by 600 days^29^. We therefore used young, six to eight-week-old heterozygotes to assess the distribution of *LacZ* as a reporter for *C9orf72* promoter activity.

**Figure 1:**
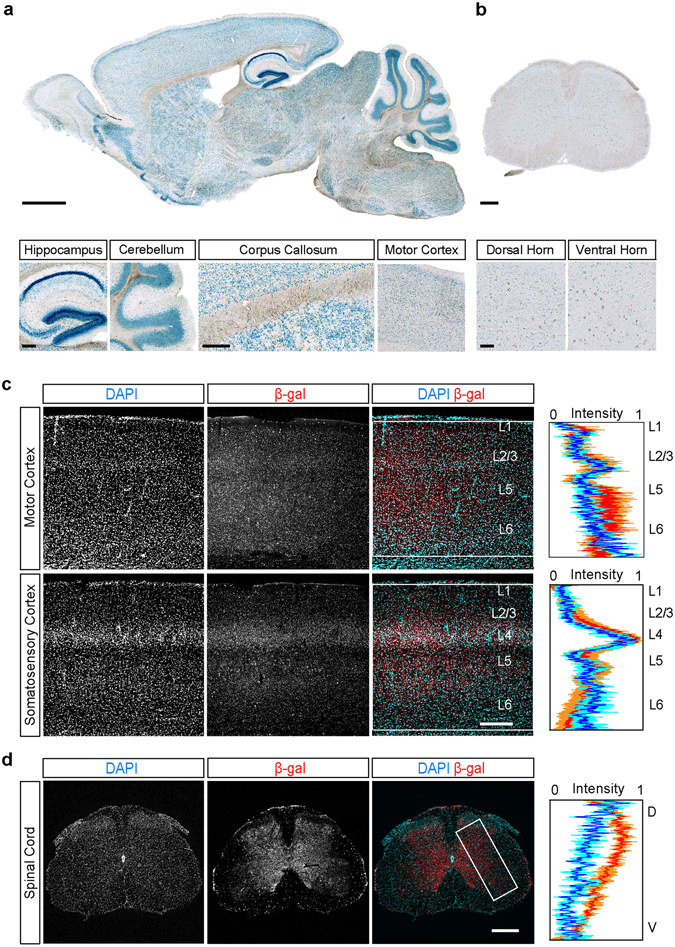
The distribution of *C9orf72* promoter activity follows overall cellular density in the central nervous system. (**a**) The overall distribution of *C9orf72* promoter activity in a parasagittal brain section from a *C9orf72* ^*LacZ/*+^ mouse revealed using X-gal staining. *C9orf72* promoter activity was high in brain regions with high cell density (dentate gyrus of the hippocampus, cerebellar granule cell layer, two left panels), low in regions with low cell density (cerebellar molecular cell layer and corpus callosum, middle panels), and medium in motor cortex (rightmost panel). (**b**) The overall distribution of *C9orf72* promoter activity in a coronal section of the lumbar spinal cord. X-gal staining was seen in both the dorsal and ventral horns (left and right panels) (**c**) Representative images showing the distribution of immunofluorescence staining for β-gal in primary motor (top) and somatosensory (bottom) cortex of *C9orf72* ^*LacZ/*+^ mice concurrently labelled with the nuclear stain, DAPI. The intensities of the β-gal (red) and DAPI (blue) signals were summed along the horizontal axis in the region indicated by the white boxes (n = 3 mice) and the intensity values plotted (mean ± SEM). (**d**) A similar analysis was performed for lumber spinal cord (*n* = 3 mice). Scale bars: (**a**) 1 mm, insets 100 µm, (**b**) 200 µm, insets 100 µm and **(c,d)** 300 µm.

We confirmed that *C9orf72* promoter activity was not limited to areas known to degenerate in ALS and FTD. X-gal staining to assess regions of β-galactosidase (β-gal) expression (encoded by *LacZ*) revealed widespread promoter activity throughout the brain and spinal cord. The regions with the most intense X-gal signals in the brain corresponded to regions with high cell density, such as the dentate gyrus of the hippocampus and the granular layer of the cerebellum (Fig. 1a). Regions with the weakest signals corresponded to areas with low cell densities such as the molecular layer of the cerebellum and the corpus callosum (Fig. 1a). We also observed broad X-gal staining in primary motor cortex (Fig. 1a) and in the spinal cord (Fig. 1b). These results suggest that the distribution of *C9orf72* promoter activity largely follows cellular density across brain regions. To more directly test this hypothesis, we compared the distribution of cells stained with the nuclear marker, DAPI, and an antibody specific to β-galactosidase (β-gal; Supplementary Fig. 1c) and found that the distribution of β-gal signal correlated with the overall cellular density across layers in primary motor cortex, primary somatosensory cortex, and spinal cord grey matter (Fig. 1c-d). Together, these results indicate that *C9orf72* promoter activity is widely distributed in the CNS, consistent with that reported in previous studies^26, 27, 32^, and largely correlates with overall cellular density.

### *The C9orf72* promoter is active in both excitatory and inhibitory neurons

**Figure 2:**
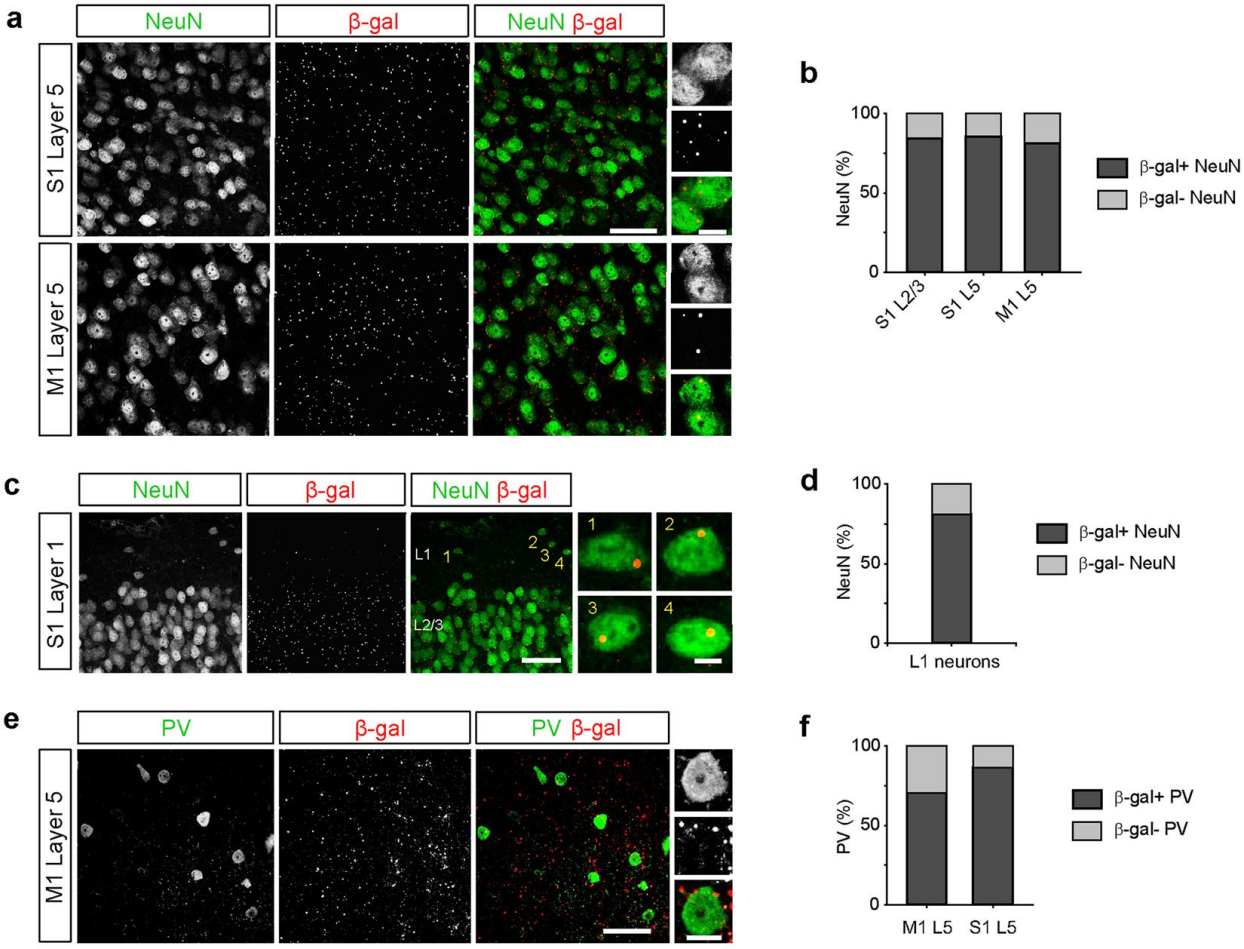
*C9orf72* promoter activity is widespread in inhibitory neurons. (**a**) Representative images of layer 5 (L5) of primary somatosensory cortex (S1, *top*) and primary motor cortex (M1, *bottom*) from *C9orf72* ^*LacZ/*+^ mice stained for NeuN (green) and β-galactosidase (β-gal; red). High magnification images show β-gal-positive puncta within NeuN-positive neurons (*rightmost panels*). Scale bars: 50 μm and 10 μm. (**b**) The percentage of neurons containing β-gal puncta in layer 2/3 and L5 of somatosensory cortex and L5 of motor cortex (S1 L2/3: 362 of 429 cells, 84.4%; S1 L5, 499 of 583 cells, 85.6%; M1 L5: 425 of 523 cells, 81.3%; *n* = 3 mice for each group, *p* = 0.1378, Chi-Square test). (**c**) Representative images of layer 1 (L1) of somatosensory cortex stained for NeuN (green) and β-gal (red). High magnification images showing β-gal-positive puncta within L1 neurons (*rightmost panels*). Scale bars: 50 μm and 5 μm. (**d**) The percentage of L1 neurons containing β-gal puncta (26 of 32 cells, 81.3%, *n* = 3 mice). (**e**) Representative images of L5 of motor cortex stained for parvalbumin (PV, green) and β-gal (red). High magnification images show β-gal puncta within a PV interneuron (*rightmost panels*). Scale bars: 50 μm and 10 μm. (**f**) The percentage of PV interneurons containing β-gal puncta in L5 of motor cortex and somatosensory cortex (M1 L5: 36 of 51 cells, 70.6%; S1 L5: 32 of 37 cells, 86.5%; *n* = 3 mice for each group, *p* = 0.0789, Chi-Square test).

Although at a macroscopic level, *C9orf72* promoter activity was correlated with overall cellular density throughout the brain and spinal cord, enrichment or reduction of *C9orf72* promoter activity in specific regions of the neocortex or spinal cord or within specific cell types in each region would go undetected at this level of analysis. Therefore, we compared the distribution of *C9orf72* promoter activity in neurons in primary motor cortex, a cortical region which undergoes degeneration in ALS, as well as primary somatosensory cortex (see Methods). *C9orf72* promoter activity was observed in more than 80% of the neurons labelled with antibodies to NeuN in layer 5 (L5) of primary motor cortex, which contains corticospinal neurons known to degenerate in ALS (Fig. 2a,b). Unexpectedly, neurons in layer 2/3 (L2/3) of primary somatosensory cortex, which consists primarily of excitatory corticocortical projection neurons, and L5 of primary som﻿atosensory cortex, exhibited a similar distribution of *C9orf72* promoter activity (Fig. 2a,b). Due to the high density of small β-gal-positive puncta in our samples, it is possible that we detected *C9orf72* promoter activity in such a large number of neurons spuriously. To assess whether the distribution of the β-gal signal was specific to these neurons, we compared the percentage of β-gal-positive neurons detected before and after inverting the β-gal channel relative to the NeuN channel in each analyzed image. Following channel inversion, the percentage of β-gal-positive neurons significantly decreased, indicating that the relationship between the distribution of β-gal-positive puncta and the distribution of neurons is greater than would be expected by chance (Supplementary Fig. 2). These data demonstrate that the overall distribution of *C9orf72* promoter activity in neurons is similar between primary motor cortex and primary sensory cortex when specific cell types are not taken into account.

Although *C9orf72* transcripts have been identified in excitatory cortical projection neurons and spinal motor neurons^26, 32^, whether *C9orf72* promoter activity is found in inhibitory neurons is not known. To test for *C9orf72* promoter activity in inhibitory neurons, we first analysed neurons in layer 1 (L1) of the cortex, which are overwhelmingly GABAergic^36, 37^. We found that β-gal-positive puncta were detected in more than 80% of L1 inhibitory interneurons (Fig. 2c-d). Similarly, we found that *C9orf72* promoter activity was detected in approximately 70 to 85% of cortical parvalbumin-expressing (PV) inhibitory interneurons, the most common type of interneurons in the cortex^37^ (Fig. 2e,f), demonstrating that *C9orf72* promoter activity is not limited to glutamatergic cortical projection neurons. Taken together, these data demonstrate that *C9orf72* promoter activity is also widespread in inhibitory interneurons.

### *C9orf72* promoter activity is enriched in corticospinal and spinal motor neurons

**Figure 3:**
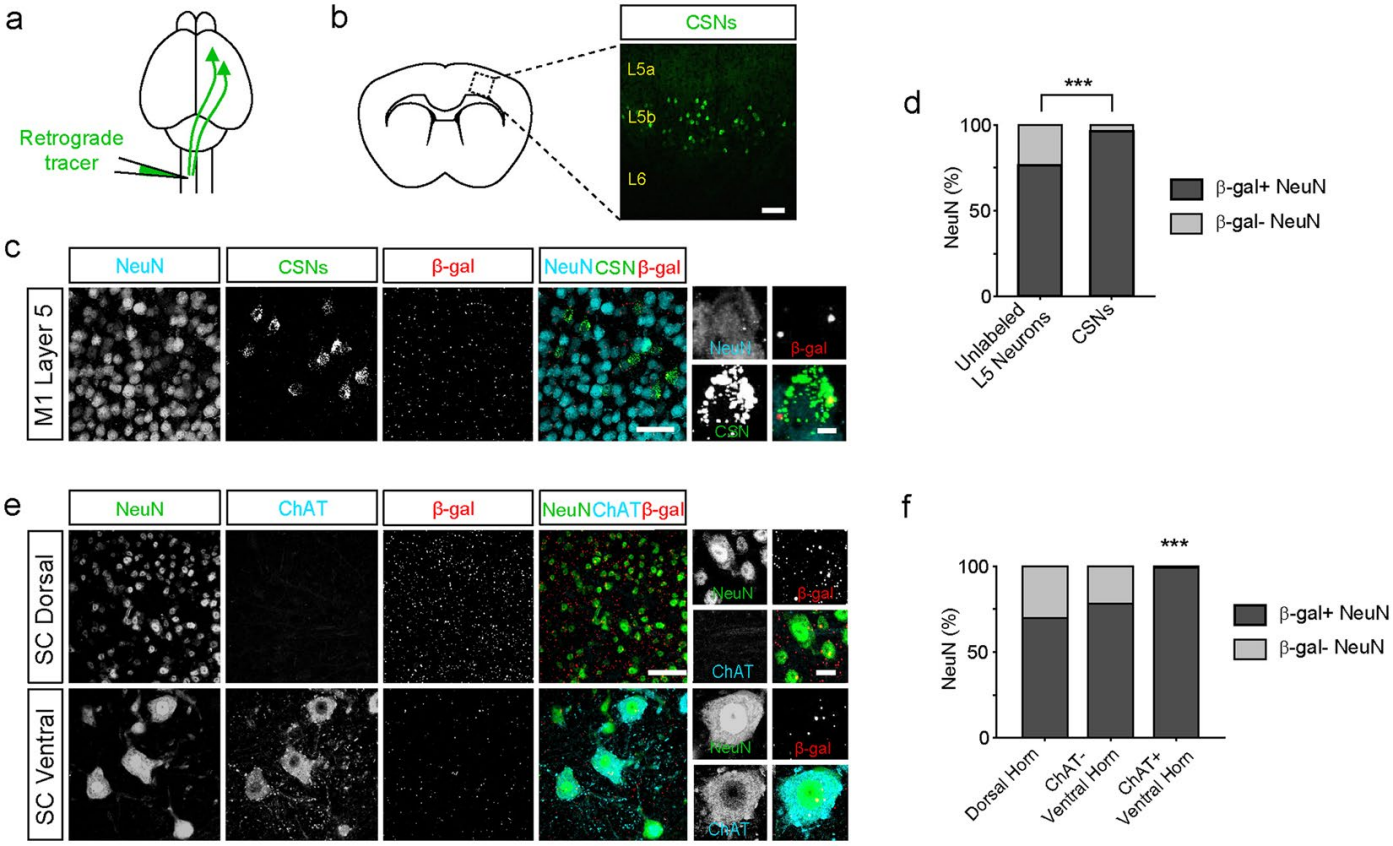
*C9orf72* promoter activity is enriched in corticospinal and spinal motor neurons. (**a**) Schematic of the experimental design. Green fluorescent retrograde tracers were injected into the contralateral cervical spinal cord to retrogradely label corticospinal neurons in *C9orf72* ^*LacZ/*+^mice. (**b**) Retrogradely labelled corticospinal neurons are shown in layer 5 (L5) of primary motor cortex. Scale bar: 100 μm. (**c**) Representative images of L5 of motor cortex showing NeuN-positive neurons (cyan), retrogradely labelled corticospinal neurons (green) and β-gal staining (red). High magnification images show β-gal puncta within a corticospinal neuron (*rightmost panels*). Scale bars: 50 μm and 5 μm. (**d**) The percentage of unlabeled NeuN-positive neurons and of corticospinal neurons (CSNs) containing β-gal puncta in L5 of motor cortex (Unlabeled NeuN-positive neurons: 1357 of 1771 cells, 76.6%; CSNs: 132 of 137 cells, 96.4%; *n* = 5 mice for each group, *p* < 0.0001, Chi-Square test). Unlabeled NeuN-positive neurons and retrogradely labelled corticospinal neurons were analyzed from the same images. (**e**) Representative images of the dorsal (*top*) and ventral horn (*bottom*) of the spinal cord immunostained for NeuN (green), Choline acetyltransferase (ChAT; cyan), and β-gal (red). High magnification images show β-gal puncta within NeuN-positive neurons in the dorsal horn (*top right)* and a ChAT-positive neuron in the ventral horn (*bottom right*). Scale bars: 50 μm and 10 μm. (**f**) The percentage of ChAT-negative and ChAT-positive neurons containing β-gal puncta in the dorsal and ventral horn of spinal cord (dorsal neurons: 734 of 1050 cells, 69.9%; ventral ChAT-negative neurons: 61 of 78 cells, 78.2%; ventral ChAT-positive neurons: 113 of 114 cells, 99.1%; *n* = 5 mice for each group; *p* < 0.0001, Chi-Square test).

The hallmark of ALS is progressive degeneration of corticospinal and spinal motor neurons. A previous study reported that the majority of layer 5 (L5) cortical neurons exhibiting *C9orf72* promoter activity were CTIP2-positive^32^, suggesting that sub-cerebral projection neurons, which include the corticospinal neurons (CSNs) that degenerate in ALS, express *C9orf72*. To further test this hypothesis, we compared the distribution of *C9orf72* promoter activity in retrogradely labelled CSNs and in unlabeled NeuN-positive neurons intermingled within L5 of motor cortex (Fig. 3a,b). A significantly higher percentage of CSNs exhibited β-gal-positive puncta than unlabeled neighboring neurons, indicating that *C9orf72* promoter activity is specifically enriched in corticospinal neurons (Fig. 3c,d; *p* < 0.0001, Chi-Square test). Similarly, a significantly higher percentage of choline acetyltransferase (ChAT)-immunoreactive spinal motor neurons exhibited detectable *C9orf72* promoter activity than either ChAT-immunonegative neurons intermingled within the same region of the ventral horn or ChAT-immunonegative neurons in the dorsal horn of the spinal cord (Fig. 3e,f; *p* < 0.0001, Chi-Square test). Together, these results indicate that *C9orf72* promoter activity is specifically enriched in both retrogradely labelled corticospinal neurons and cholinergic spinal motor neurons, neuronal types that are specifically vulnerable in ALS.

### *C9orf72* promoter activity in Purkinje cells and cerebellar granule cells

**Figure 4:**
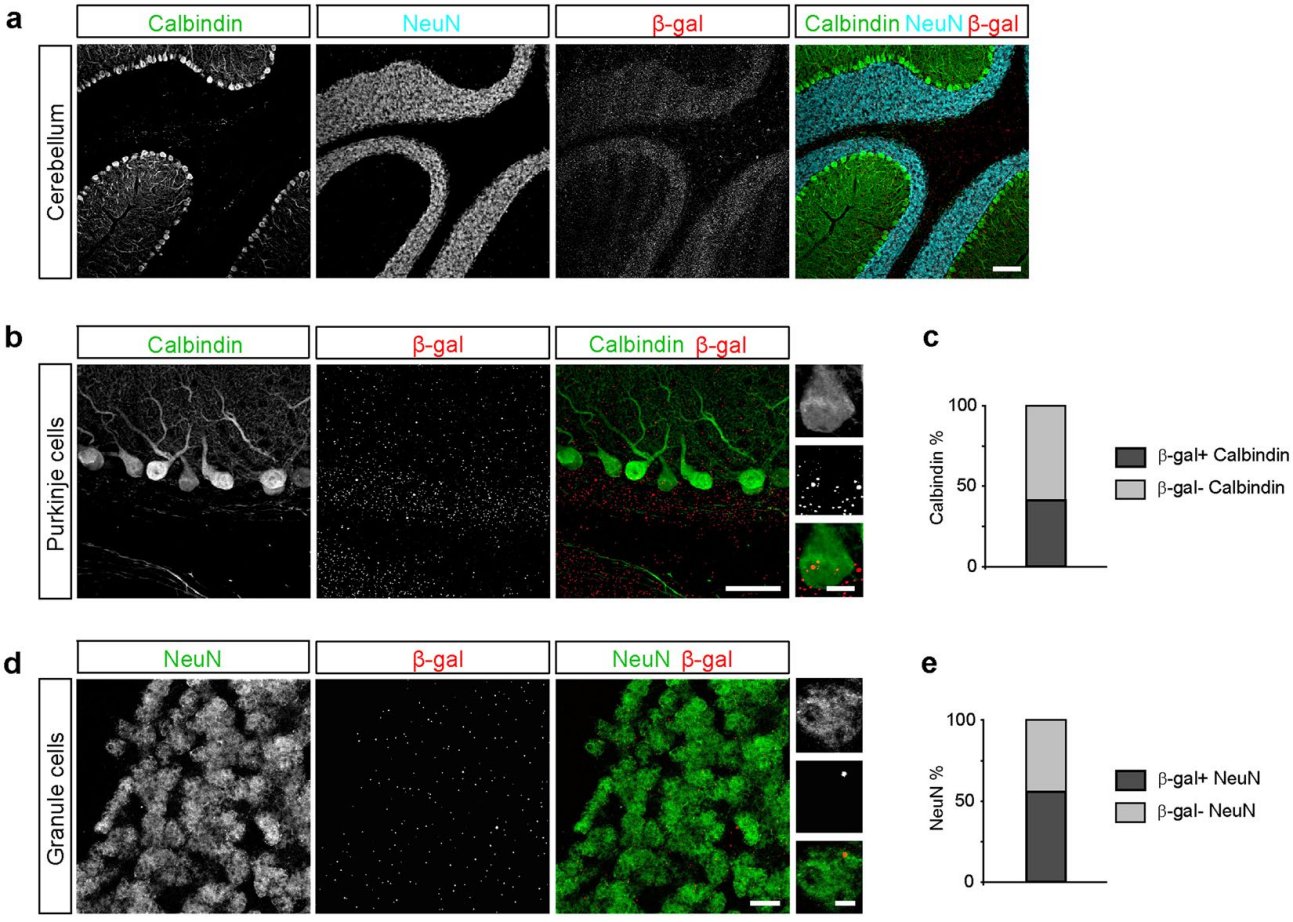
*C9orf72* promoter activity in Purkinje cells and granule cells of the cerebellum. (**a**) Representative images of the cerebellum showing calbindin-positive Purkinje cells (green), NeuN-positive granule cells (cyan), and β-gal staining (red). Scale bar: 100 μm. (**b**) Representative images of calbindin-positive Purkinje cells (green) and β-gal (red). Scale bars: 50 μm and 10 μm. (**c**) The percentage of Purkinje cells containing β-gal puncta (35 of 85 cells, 41.2%, *n* = 3 mice). (**d**) Representative images of NeuN-positive granule cells (green) and β-gal (red). Scale bars: 10 μm and 2 μm. (**e**) The percentage of granule cells containing β-gal puncta: (306 of 548 cells, 55.8%; *n* = 3 mice).

*C9orf72*-mediated disease is notable for RNA foci and neuronal inclusions found in the cerebellum^38–41^. RAN-translated polypetides stemming from the hexanucleotide repeat expansion have been detected in Purkinje cells and granule cells^40^. Furthermore, prior studies have detected high levels of *C9orf72* expression in the cerebellum^23, 42^. Here we assessed *C9orf72* promoter activity in both Purkinje cells and granule cells in the cerebellum. Consistent with the X-gal staining showing intense signal in the cerebellum (Fig. 1a), we detected *C9orf72* promoter activity in a large percentage of both Purkinje cells and cerebellar granule cells (Fig. 4). Overall, 41.2% of Purkinje cells and 55.8% of granule cells had detectable *C9orf72* promotor activity (*p* = 0.0116, Chi-Square test), indicating that *C9orf72* promoter activity is relatively enriched in the granule cell layer. These data provide additional evidence for cell-type specific enrichment of *C9orf72* promoter activity in distinct cell types in affected brain regions in *C9orf72*-mediated disease.

### Cell-type and region specific regulation of *C9orf72* promoter activity in glial cells

**Figure 5:**
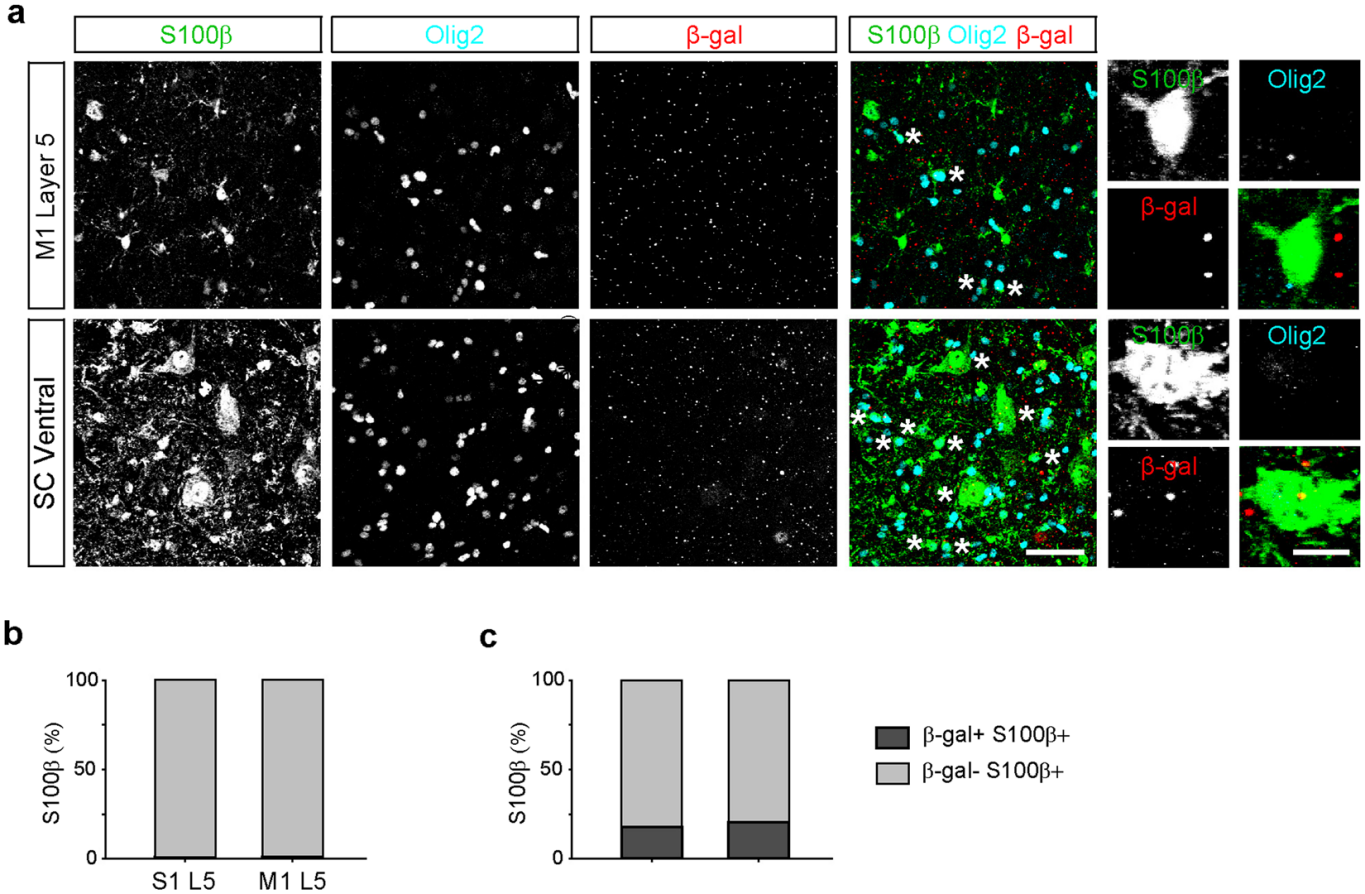
Differential *C9orf72* promoter activity in cortical and spinal cord astrocytes. (**a**) Representative images of layer 5 (L5) in primary motor cortex (M1, *top*) and the ventral horn of the spinal cord (SC, *bottom*) of *C9orf72* ^*LacZ/*+^ mice immunostained for S100β-positive astrocytes (green), Olig2-positive oligodendrocyte lineage cells (cyan), and β-gal (red). Asterisks indicate S100β-positive Olig2-positive oligodendrocyte lineage cells and S100β-positive motor neurons which were excluded from the analysis. High magnification images show an astrocyte in motor cortex which lacks β-gal puncta (*top right*) and astrocytes in the spinal cord which contain β-gal puncta (*bottom right*). Scale bars: 50 μm and 10 μm. Percentage of astrocytes containing β-gal puncta in L5 of primary motor and primary somatosensory cortex (**b**) L5 motor cortex: 1 of 92 cells, 1.1%, L5 somatosensory cortex, 1 of 98 cells, 1.0%, *n* = 3 mice for each group, *p* = 0.9641, Chi-Square test) and spinal cord (**c**) dorsal spinal cord: 15 of 84 cells, 17.9%, ventral spinal cord: 16 of 78 cells, 20.5%, *n* = 3 mice for each group, *p* = 0.6676, Chi-Square test).

Glial cells play important roles in the pathophysiology of ALS. Astrocytes undergo reactive changes in ALS, although their role in the initiation and progression of *C9orf72*-mediated ALS remains to be elucidated^17, 43–45^. While a prior study reported that spinal cord astrocytes did not express *C9orf72* ^32^, RNA sequencing experiments detected low levels of *C9orf72* transcripts in mouse^28, 46^ and human astrocytes^47^. Consistent with the RNA sequencing data, we found that approximately 20% of S100β-positive astrocytes in the spinal cord had detectable *C9orf72* promoter activity (Fig. 5a-c). Interestingly, almost no S100β-positive astrocytes in the cortex exhibited detectable *C9orf72* promoter activity (Fig. 5a-c). These data indicate that far fewer astrocytes than neurons express *C9orf72*, and that *C9orf72* promoter activity is not only regulated in a cell-type specific manner but also in a region dependent way.

**Figure 6:**
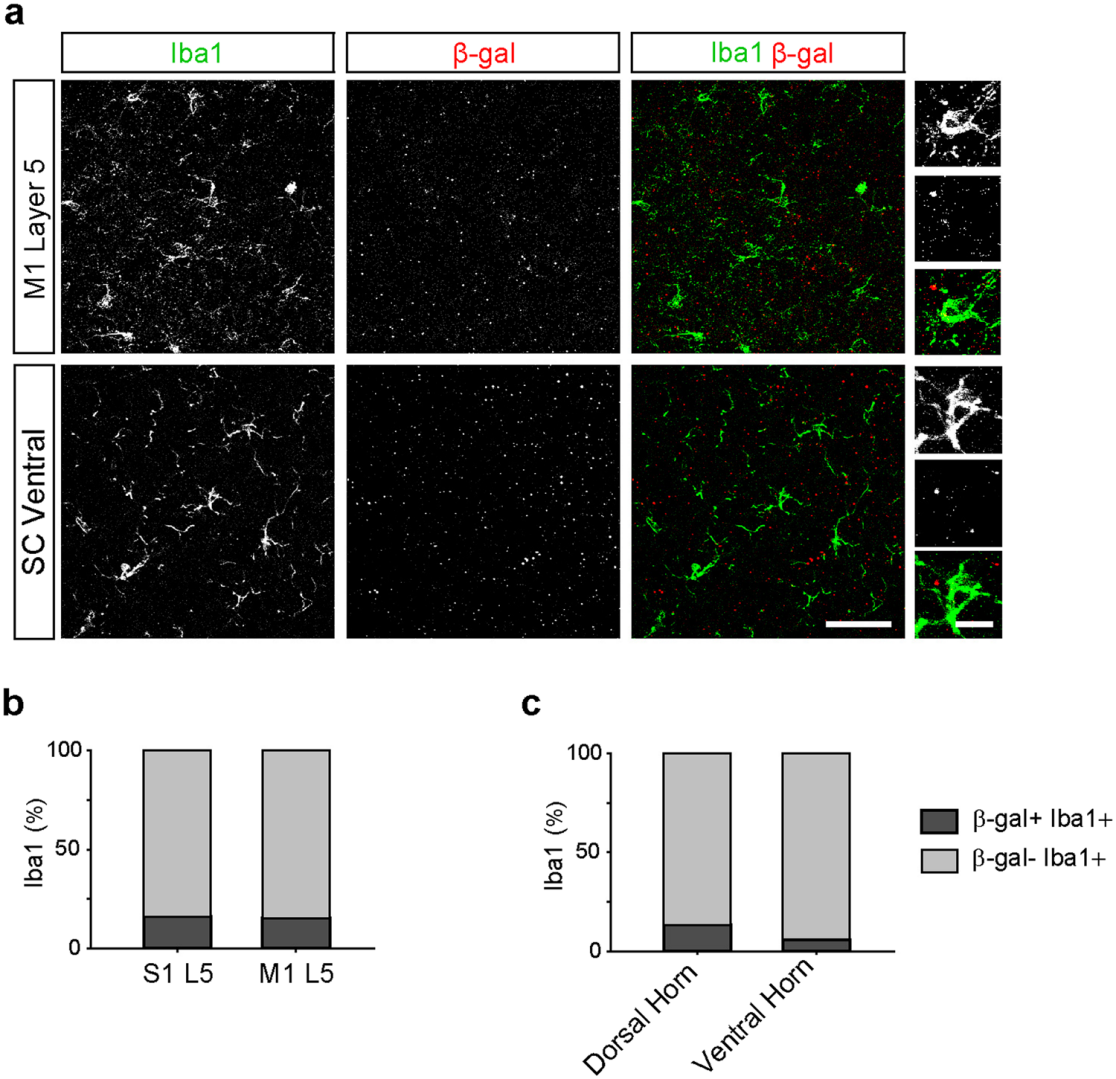
*C9orf72* promoter activity is detected in few microglia. (**a**) Representative images of layer 5 (L5) of primary motor cortex (M1, *top*) and the ventral horn of the spinal cord (SC, *bottom*) of *C9orf72* ^*LacZ/*+^ mice immunostained for Iba1-positive microglia (green) and β-gal (red). High magnification images show microglia which do not contain β-gal puncta in the motor cortex (*top right*) and the spinal cord (*bottom right*). Scale bars: 50 μm and 10 μm. The percentage of microglia containing β-gal puncta in L5 of the primary motor and primary somatosensory cortex (**b**) L5 of somatosensory cortex: 16 of 127 cell, 16.0%; L5 of motor cortex: 22 of 160 cells, 15.2%; *n* = 7 mice for each group, *p* = 0.7749, Chi-Square test) and the spinal cord (**c**; dorsal spinal cord: 14 of 106 cells, 13.2%; ventral spinal cord: 2 of 34 cells, 5.9%, *n* = 3 mice for each group, *p* = 0.2427, Chi-Square test).

Recent studies have demonstrated that *C9orf72* plays an important role in regulating the immune system^24, 25, 28^ and have reported high levels of *C9orf72* transcript expression in bulk cellular populations enriched for microglia^28, 46^. In contrast, a prior study detected *C9orf72* promoter activity in only a small subset of microglia in the anterior horn of the spinal cord^32^. Similarly, we found that only a small proportion of Iba1-immunoreactive microglia in the cortex and spinal cord had detectable *C9orf72* promoter activity (Fig. 6a–c). These findings suggest that the level of expression of *C9orf72* varies substantially across the population of microglial cells in the brain and spinal cord, with only a few cells expressing high levels and the majority expressing low or undetectable levels.

**Figure 7:**
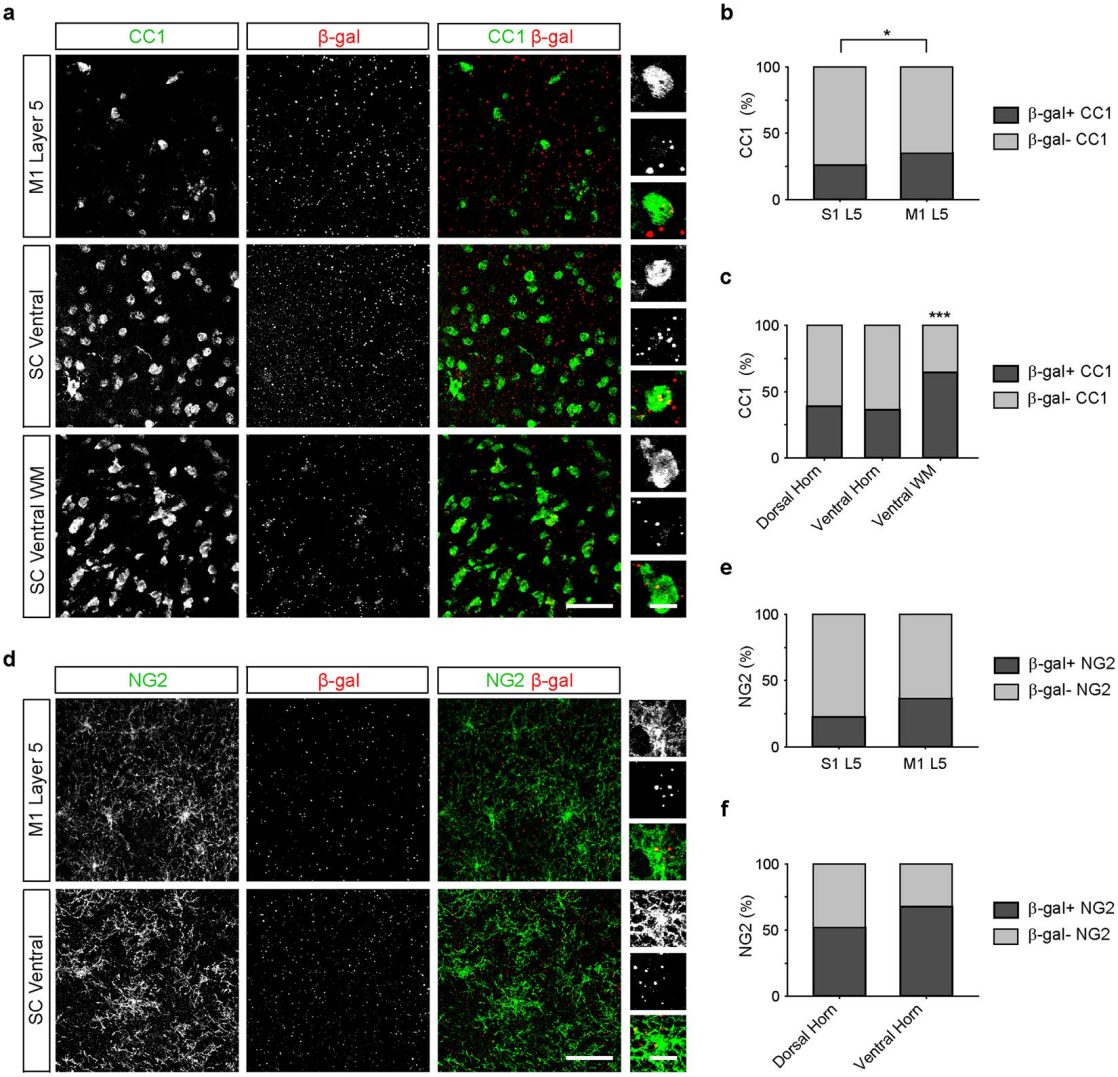
*C9orf72* promoter activity in oligodendrocytes and OPCs is enriched in cortical regions associated with neurodegeneration in amyotrophic lateral sclerosis. (**a**) Representative images of layer 5 (L5) of the primary motor cortex (M1, top), ventral horn of the spinal cord (SC, middle), and the ventral white matter of the spinal cord (SC WM, bottom) of *C9orf72* ^*LacZ/*+^ mice immunostained for CC1-positive oligodendrocytes (green) and β-gal (red). High magnification images show colocalization of β-gal puncta within oligodendrocytes (right panels).The percentage of oligodendrocytes containing β-gal puncta in L5 of primary somatosensory (S1) and motor (M1) cortex (**b**) S1 L5: 38 of 167 cells, 26.0%; M1 L5: 82 of 234 cells, 35.0%; n = 5 mice for each group, p = 0.0140, Chi-Square test) and spinal cord (**c**) dorsal spinal cord: 209 of 533 cells, 39.2%; ventral spinal cord: 191 of 525 cells, 36.4%; spinal cord white matter: 283 of 438 cells, 64.6%, n = 3 mice for each group, p < 0.0001 Chi-Square) (**d**) Representative images of L5 of primary motor cortex (M1, top) and the ventral horn of the spinal cord (bottom) immunostained for NG2-positive oligodendrocyte precursor cells (OPCs; green) and β-gal (red). High magnification images show colocalization of β-gal puncta within oligodendrocytes (right panels). The percentage of OPCs containing β-gal puncta in L5 of primary motor (M1) and primary somatosensory (S1) cortex (**e**) S1 L5: 21 of 92 cells, 22.6%; M1 L5: 33 of 92 cells, 36.5%; n = 5 mice for each group, p = 0.0520, Chi-Square test) and spinal cord (**f**) dorsal spinal cord: 39 of 75 cells, 52.0%; ventral spinal cord: 44 of 65 cells, 67.7%; n = 3 mice for each group, p = 0.0594, Chi-Square test). Scale bars: 50 μm and 10 μm.

Oligodendrocytes provide metabolic support critical for neuronal health particularly for long axons. The degeneration of oligodendrocytes seen in ALS patient tissue and in mouse models of the disease suggests that these cells play an important role in the selective vulnerability of specific neuronal populations in ALS^48–51^. Removal of the ALS mutation *SOD1* ^*G37R*^ from the oligodendrocyte lineage significantly delays disease onset and extends the lifespan of ALS model mice^52^, providing further support for an important contribution of oligodendrocyte dysfunction in the disease. Although *C9orf72* expression has been reported in oligodendrocytes^26, 28^, the distribution of *C9orf72* promoter activity in the cortex and spinal cord is still poorly understood. We found that more than 25% of CC1-immunoreactive oligodendrocytes in layer 5 of primary somatosensory cortex and of primary motor cortex exhibited *C9orf72* promoter activity. Interestingly, we detected β-gal puncta in a significantly greater percentage of CC1-positive oligodendrocytes in motor cortex than primary somatosensory cortex (Fig. 7a,b; *p* = 0.014, Chi-Square test), indicating enrichment of *C9orf72* expression in cortical regions which undergo the most profound degeneration in ALS. An even greater percentage of oligodendrocytes in the spinal cord exhibited detectable *C9orf72* promoter activity (Fig. 7a,c); the highest percentage of β-gal-positive, CC1-positive oligodendrocytes was found in the ventral white matter of the spinal cord, where almost 65% of oligodendrocytes were positive for β-gal (Fig. 7c; *p* < 0.0001, Chi-Square test). *C9orf72* promoter activity was also observed in approximately 20–35% of cortical NG2-immunoreactive oligodendrocyte precursor cells (OPCs; Fig. 7d,e). More than 50% of OPCs of the spinal cord also exhibited *C9orf72* promoter activity (Fig. 7d﻿,f). These data indicate that *C9orf72* promoter activity is detected in a much larger percentage of oligodendrocytes and OPCs than in astrocytes and microglia, and that *C9orf72* promoter activity is specifically enriched in oligodendrocytes in regions thought to degenerate in ALS.

## Discussion

Genes that are mutated in ALS are widely expressed in the CNS, creating unique vulnerabilities in distinct cell types. To understand how these mutations alter cellular physiology we must define both the tissue and cellular expression patterns of these mutant genes. Here, we show that although the distribution of *C9orf72* promoter activity follows overall cellular density, there is striking enrichment in neuronal and glial cell types that degenerate in ALS. *C9orf72* promoter activity was detected in a significantly higher percentage of both corticospinal and spinal motor neurons than in neighboring neuronal cell types. Similarly, we demonstrate that *C9orf72* promoter activity was enriched in oligodendrocytes in cortical regions associated with ALS pathology compared to unaffected areas. In contrast to neurons and oligodendrocytes, we detected *C9orf72* promoter activity in few astrocytes and microglia^32^. Together, these data indicate that the pattern of degeneration seen in ALS reflects the distribution of *C9orf72* promoter activity, suggesting that cell autonomous effects in these populations of neurons and glia may account for their loss.

The widespread expression of *C9orf72* promoter activity we observed in this Neo-deleted KOMP mouse is consistent with the overall expression patterns reported in recent studies of the KOMP mouse^26, 32^ as well as *C9orf72* RNA expression data^26–28, 53^, and indicates that the distribution of *C9orf72* promoter activity grossly follows cellular density throughout the CNS. However, an analysis of specific cell types demonstrated that widely varying fractions of cells expressed *C9orf72*. For example, a large majority of both inhibitory and excitatory neurons exhibited *C9orf72* promoter activity while a small minority of astrocytes did. The widespread expression of *C9orf72* by interneurons which we demonstrate here is particularly interesting, given their pivotal role in regulating neuronal excitability and brain states, and dysfunction of cortical inhibitory neurons may contribute to the cortical hyperexcitability seen in patients with *C9orf72* disease^54, 55^.

Our data also indicate that *C9orf72* promoter activity is enriched in cell types and brain regions that undergo degeneration in ALS. A higher percentage of corticospinal neurons express *C9orf72* than either neighboring neurons in motor cortex or neurons in L5 of somatosensory cortex. Similarly, a higher percentage of oligodendrocytes in L5 of motor cortex express *C9orf72* than in L5 of somatosensory cortex. We also found that approximately 25–50% of oligodendrocytes and OPCs in the cortex and spinal cord expressed *C9orf72*. Importantly, we show that a greater fraction of oligodendrocytes in L5 of motor cortex expresses *C9orf72*, providing further evidence that cell types that degenerate in ALS express *C9orf72* more widely than other cell types in the CNS. Together with the evidence of widespread degeneration of oligodendrocytes in ALS patient tissue and mouse models^48–51^, and the finding that removal of the *SOD1* ^*G37R*^ ALS mutant transgene selectively from oligodendrocyte lineage cells significantly delays disease onset and extends the lifespan of ALS model mice^52^, these data support the hypothesis that oligodendroglia are critical primary players in the selective degeneration of CNS regions in ALS. The pathological mechanisms associated with expression of mutated *C9orf72* may confer selective vulnerability on these cell types during the course of ALS and may help to explain the regional pattern of degeneration observed in ALS.

A hallmark of *C9orf72*-mediated disease is cerebellar pathology including RNA foci and neuronal inclusions^38–41^. Prior studies have also detected high levels of *C9orf72* expression in the cerebellum^23, 42^. Consistent with this work, we detected *C9orf72* promoter activity in both Purkinje cells and cerebellar granule cells. Interestingly, a higher percentage of granule cells exhibited detectable *C9orf72* promoter activity than neighboring Purkinje cells, contributing to the intense staining we detected in the granule cell layer. Although there are marked neuropathological findings in the cerebellum in *C9orf72*-mediated disease and high levels of *C9orf72* expression, the relationship between these findings and any neurodegeneration remains unclear^38, 56^.

In contrast to the enrichment of *C9orf72* promoter activity that we detected in neurons and oligodendrocyte lineage cells, we found that very few astrocytes and microglia exhibited detectable *C9orf72* promoter activity. Furthermore, there were striking regional differences in expression. For example, almost no cortical astrocytes exhibited *C9orf72* promoter activity while approximately 20% of the astrocytes in the spinal cord expressed *C9orf72*. These results are consistent with analyses of *C9orf72* transcript expression, although an early study did not detect *LacZ* expression in spinal cord astrocytes in a similar mouse line^24, 26, 28, 32, 46^. Recent studies have demonstrated high levels of *C9orf72* expression in enriched microglia cell populations^26, 28, 46^. This result is surprising in view of the small percentage of spinal cord microglia previously found to exhibit *C9orf72* promoter activity in the KOMP mouse^32^. Not only did we also detect *C9orf72* promoter activity in only a small fraction of microglia in the spinal cord, we found that few microglia in the cortex exhibited detectable *C9orf72* promoter activity. It is possible that *C9orf72* transcript levels may depend on the activation state of a microglial cell, and that *C9orf72* expression is rapidly upregulated when cells are dissociated from the brain for gene expression analysis. Recent work demonstrating that immune challenge of monocytes increases *C9orf72* expression hints at this possibility, although unchallenged monocytes express higher levels of *C9orf72* than microglia from fetal human brain tissue^23^. Alternatively, there may be widely varying levels of expression in different microglial cells, with a small fraction of microglial cells expressing high levels of *C9orf72* leading to high levels of transcript detection in bulk populations. Understanding the patterns of *C9orf72* expression in combination with the distribution of the protein in different cell types represents an essential step in elucidating the mechanisms of dysfunction in CNS cells in the spectrum of *C9orf72*-associated diseases. Several studies have demonstrated decreased *C9orf72* transcript and protein levels in patients with ALS and FTD^19–23^, suggesting that haploinsufficiency may contribute to ALS pathogenesis. Our analysis of the patterns of *C9orf72* expression suggests that the cell types that undergo degeneration in ALS, including corticospinal neurons, spinal motor neurons and oligodendrocytes, would be most affected by any *C9orf72* haploinsufficiency. However, the mechanisms underlying the range of phenotypes following *C9orf72* deletions in mouse models remains to be elucidated^24–29^, and understanding this variability remains an essential step for uncovering the cellular mechanisms underlying ALS and other *C9orf7*2-linked diseases.

## Methods

### Generation of *C9orf72*^*LacZ/*+^ mice

All experimental procedures were approved by the Johns Hopkins Animal Care and Use Committee and conducted in accordance with the guidelines of the National Institutes of Health and the Society for Neuroscience. The mice carrying a targeted deletion of *C9orf72* and insertion of *LacZ* were generated as described^29^. In brief, several embryonic stem cell lines harboring an insertion that replaced exons 2–6 of the mouse *C9orf72* gene (3110043O21Rik) with *LacZ* (National Institutes of Health Knockout Mouse Project) were used for blastocyst injection to generate chimeric mice which were then selected for germline transmission. The original embryonic stem cells had the genetic background of C57BL/6N-Atm1Brd and the derived mice were maintained on the C57BL/6 background. Male mice bearing the targeted allele were crossed with *Sox2-Cre* recombinase transgenic female mice (Jackson Laboratory, 008454), maintaining the C57BL/6 background, to remove the LoxP-flanked neomycin selection cassette in all progeny. Subsequent breeding eliminated the Sox2-Cre transgene from our mouse line. Six to eight-week-old Neo-deleted *C9orf72* ^*LacZ/*+^ heterozygous mice were used for all experiments. The genotyping primers were the following: gaatggagatcggagcacttatgg (wild-type, forward), gccttagtaactaagcttgctgccc (wild-type, reverse), gcacaagctatgttcatttgg (KO, forward), gactaacagaagaacccgttgtg (KO, reverse).

### Immunohistochemistry and X-galactosidase staining

Mice were deeply anesthetized with sodium pentobarbital (100 mg/kg) and perfused transcardially with 4% paraformaldehyde in 0.1 M sodium phosphate buffer. Brain and spinal cord tissue was isolated and postfixed in this solution overnight at 4 °C, then washed in phosphate buffer. Spinal cord tissues were cryoprotected in 30% sucrose, and sectioned at 35 µm thickness on a cryostat. Brain sections, 35 μm thick, were prepared using a vibratome (VT-1000S, Leica). To obtain sections of motor cortex, the brain was mounted in coronal orientation on a 15° ramp prior to cutting slices. To obtain sections of somatosensory cortex, the brain was mounted in parasagittal orientation on a 30° ramp prior to cutting slices. Cerebellar slices were obtained in the parasagittal orientation, after mounting the cerebellum on a flat block. Free-floating sections were permeabilized with 0.3% Triton X-100 in 0.1 M sodium phosphate buffer for at least 5 min and then blocked with 0.3% Triton X-100 and 5% normal donkey serum in 0.1 M sodium phosphate buffer (blocking solution) for at least 1 h at room temperature. When performing immunohistochemistry for calbindin, 5% normal goat serum was also added. Sections were then incubated with primary antibodies prepared in blocking solution overnight at 4 °C and then incubated with secondary antibodies in blocking solution for at least 2 h at room temperature. Primary antibodies used included the following: rabbit anti-β-galactosidase (1:5000; gift from Dr. Joshua Sanes, Harvard University, Cambridge, MA), guinea pig anti-NG2 (1:30,000; Dr. Dwight Bergles, Johns Hopkins University, Baltimore, MD), mouse anti-APC (CC1; 1:50; Millipore, Cat. No. OP80), guinea pig anti-Olig2 (1:20,000; gift from Dr. Ben Novitch, University of California, Los Angeles, CA), mouse anti-S100β (1:400; Sigma, Cat. No. S2532), goat anti-Iba1 (1:250; Novus, Cat. No. NB100–1028), mouse anti-NeuN (1:500; Millipore, Cat. No. MAB377), mouse anti-Parvalbumin (1:300; Swant, Cat. No. PV235), goat anti-ChAT (1:500; Millipore, Cat. No. AB144P) and chicken anti-calbindin D28 (1:100, EnCor Biotechnology Inc., Cat. No. CPCA-Calb). Secondary antibodies used, all raised in donkey except for the goat anti-chicken secondaries, included the following: Alexa Fluor 488-, 546-, and 647- as well as Cy2-, Cy3-, and Cy5-conjugated secondary antibodies to rabbit, guinea pig, mouse, and goat (1:2000; Invitrogen and Jackson ImmunoResearch). For X-galactosidase staining, sections were incubated in 1 mg/mL X-gal (Invitrogen) in a solution of 5 mM potassium ferricyanide (Sigma), 5 mM potassium hexacyanoferrate(II) trihydrate (Sigma), and 2 mM magnesium chloride (Sigma) in PBS for 24 h at 37 °C.

### Corticospinal neuron identification with retrograde neuronal tracers

To identify corticospinal neurons, retrograde neuronal tracers were injected into the spinal cord. Mice were anesthetized with ketamine (50 mg/kg), dexmedetomidine (25 μm/kg) and the inhalation anesthetic, isoflurane (0.5–3%). A small laminectomy was performed over the left cervical cord. After removing one vertebra (C5-C6), 100–200 nl of retrograde neuronal tracer, either green Retrobeads (Lumafluor) or Alexa Fluor 488-conjugated cholera toxin B (Invitrogen), were pressure injected through a glass pipet (20–30 μm tip, inner diameter, Drummond), 500 μm lateral to the midline of the exposed spinal cord at a depth of 1000 μm. Buprenorphine (0.05 mg/kg) was administered to all animals post-operatively as an analgesic. Mice were sacrificed 7–12 days after tracer injections and brain sections were cut and processed as described above.

### Image acquisition and analysis

To assess the overall distribution of *C9orf72* promoter activity, fluorescence images were collected on an AxioImager M1 microscope (Carl Zeiss). Intensity plots for DAPI, β-gal, and ChAT were made by averaging the signal intensity along the horizontal axis using ImageJ software (NIH). To analyze the distribution of *C9orf72* promoter activity within cell types, fluorescence images were acquired with an LSM 510 Meta confocal microscope (Carl Zeiss). Stacks of confocal images (0.3 µm *z*-interval) were imported into Imaris software for three-dimensional analysis. Surface renderings of NeuN-positive, ChAT-positive, PV-positive, calbindin-positive, Iba1-positive, CC1-positive, NG2-positive, and S100β-positive/Olig2-negative cells were created, the locations of β-gal-positive puncta were marked using the Spots function, and any cell with one or more β-gal-positive puncta within the three-dimensional rendering was counted as exhibiting *C9orf72* expression. As the cerebellar granule cells were very tightly packed, there were occasionally small groups of NeuN-positive neurons that could not be clearly segmented into individual neurons. These groups were eliminated from the analysis. To determine whether the distribution of β-gal puncta related to the underlying cellular distribution, the channel containing the β-gal signal was flipped horizontally while leaving the cell type-specific channels unchanged. The same counting procedure was then applied to this new configuration.

### Analysis of gene expression

The transcript analysis was performed using RNA-sequencing (RNA-seq) data from a previously published study^28^. The RNA-seq data were derived from spinal cords of 3-month old wild-type and *C9orf72* (3110043O21Rik) knockout mice (three animals from each group). These knockout mice and the independent line analyzed in the present study were generated from a common source of mouse embryonic stem cells (DEPD00552, the Mouse Biology Program, www.mousebiology.org). The RNA-seq reads were aligned to mouse genome build mm10 using HISAT2 using standard parameters^57^. The BAM files were then visualized using integrated genome viewer^58^. The Sashimi plot was generated to visualize the RNA alignment as well as the splice junctions with a minimum junction coverage of 2.

### Statistics

The Chi-Square test was used to determine whether there was a significant difference in the expected frequencies of β-gal immunoreactive cells between cell types or between different regions. For the flipped image test, a paired *t-*test was used after confirming the normality of data distribution with the Shapiro Wilk normality test. *p* < 0.05 was considered to be statistical significant, and the asterisk was used to mark the statistical significance on the graphs, * for *p* < 0.05, ** for *p* < 0.01, and *** for *p* < 0.001.

## Electronic supplementary material

Supplementary information
