# Supplementary material for: Cell-type specific differences in promoter activity of the ALS-linked C9orf72 mouse ortholog
Source: Sci Rep. 2017 Jul 18;7:5685. doi: 10.1038/s41598-017-05864-2 (PMC5515847; doi:10.1038/s41598-017-05864-2)
Supplement: Supplementary file 1 — Supplementary information [file 41598_2017_5864_MOESM1_ESM.pdf]

Supplementary Information

Cell-type specific differences in promoter activity of the ALS-linked *C9orf72* mouse ortholog

Abraham J. Langseth[1,‡], Juhyun Kim[1,‡], Janet E. Ugolino[1,2], Yajas Shah[1,2], Ho-Yon Hwang[1,2], Jiou Wang[1,2,*], Dwight E. Bergles[1,*], Solange P. Brown[1,*]

Affiliations:

[1]Solomon H. Snyder Department of Neuroscience, Johns Hopkins University School of Medicine, Baltimore, Maryland, 21205.

[2]Department of Biochemistry and Molecular Biology, Bloomberg School of Public Health, Johns Hopkins University, Baltimore, Maryland 21205

[‡]These authors contributed equally.

*Corresponding authors: Solange P. Brown (spbrown@jhmi.edu), Dwight E. Bergles (dbergles@jhmi.edu), Jiou Wang (jiouw@jhmi.edu).

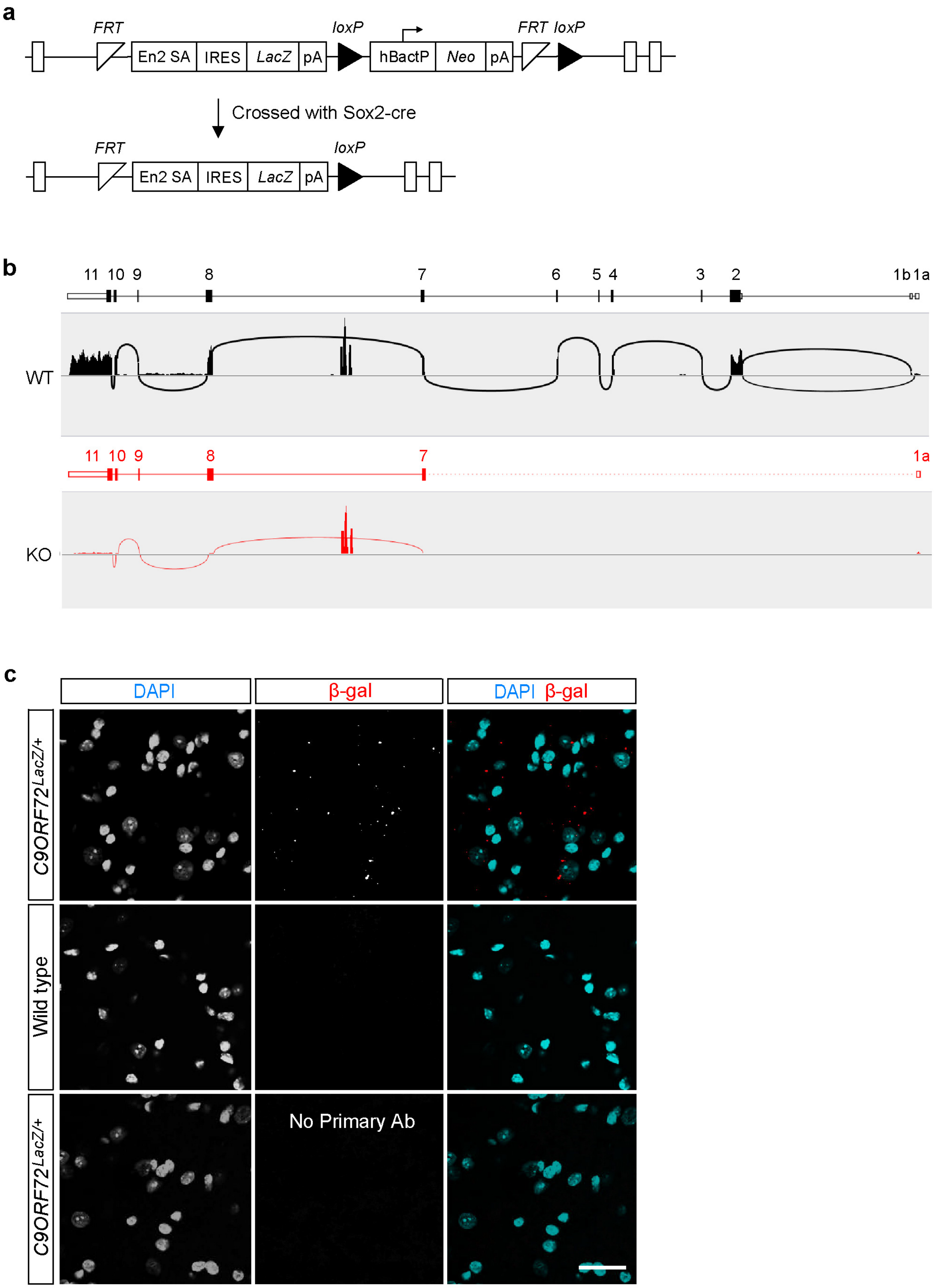

a
FRT
En2 SA
IRES
LacZ
pA
loxP
hBactP
Neo
pA
FRT loxP
Crossed with Sox2-cre
b
11 10 9 8 7 6 5 4 3 2 1b 1a
WT
KO
c
DAPI
β-gal
DAPI β-gal
C9ORF72 LacZ/+
Wild type
No Primary Ab

Supplementary Figure 1. Genetic scheme of *Neo*-deleted *C9orf72*$^{LacZ/+}$ mouse lines and validation of antibodies used to detect β-galactosidase-positive puncta in *C9orf72*$^{LacZ/+}$ mice. (**a**) *C9orf72*$^{LacZ/+}$ mice were generated from ES cells obtained from the National Institutes of Health Knockout Mouse Project (*311004O21Rik*) on a C57BL/6 background. The *Neo* cassette was removed by breeding founders to Sox2-Cre mice on a C57BL/6 background and the Sox2-Cre transgene was subsequently eliminated with further breeding. For further details, see[29]. (**b**) Sashimi plot of RNA-seq analysis of spinal cord samples from wild-type and knockout mice at the *3110043O21Rik* locus[28]. In the wild-type mice, the RNA alignment data along with splice junction tracks are shown, supporting a transcript model of 11 exons with two alternative starts. In the homozygous knockout mice, exons 2-6 are absent from the transcript, in accordance with the targeted allele. The transcript level appears to be lower in the knockout mice than in the wild-type control, and all detected transcripts start from exon 1a. (**c**) Representative images of a spinal cord section from a *C9orf72*$^{LacZ/+}$ mouse (*top*) immunostained for β-galactosidase (β-gal; red) and DAPI (blue) showing β-gal-positive puncta. Representative images from a wildtype mouse (*middle*) immunostained for β-gal (red) and DAPI (blue) showing no background staining. Representative images from a *C9orf72*$^{LacZ/+}$ mouse (*top*) immunostained by omitting the primary antibody for β-gal (red) and DAPI (blue). No β-gal-positive puncta were detected in the wild type mice or in experiments in which the primary antibody was omitted. Scale bar, 30 µm.

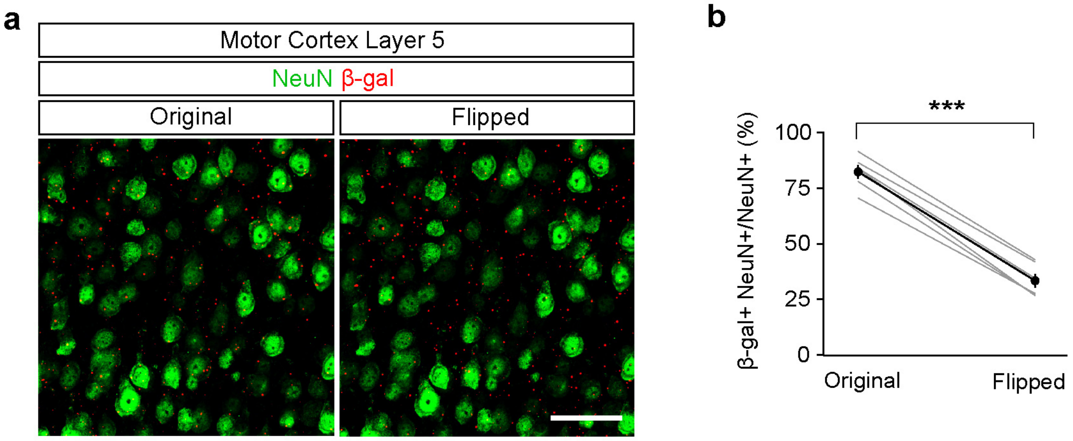

Supplementary Figure 2. The localization of the β-galactosidase-positive puncta is specific to cortical neurons. Representative images of layer 5 of motor cortex from *C9orf72*$^{LacZ/+}$ mice immunostained for NeuN and β-galactosidase (β-gal) before (**a;** left) and after (**a;** right) the β-gal channel was flipped around the vertical axis relative to the NeuN channel. Scale bar, 50 µm. (**b**) Quantification of the colocalization of β-gal-positive puncta in NeuN-positive neurons in the original and flipped configurations (Original: 82.4 ± 3.0%; Flipped: 33.5 ± 3.1%, n = 6 images, p < 0.0001, paired t-test).
